# Novel Parvovirus and Related Variant in Human Plasma

**DOI:** 10.3201/eid1201.050916

**Published:** 2006-01

**Authors:** Jacqueline F. Fryer, Amit Kapoor, Philip D. Minor, Eric Delwart, Sally A. Baylis

**Affiliations:** * National Institute for Biological Standards and Control, Hertfordshire, United Kingdom;; † Blood Systems Research Institute, San Francisco, California, USA;; ‡ University of California, San Francisco, California, USA

**Keywords:** Parvovirus, plasma

## Abstract

We report a novel parvovirus (PARV4) and related variants in pooled human plasma used in the manufacture of plasma-derived medical products. Viral DNA was detected by using highly selective polymerase chain reaction assays; 5% of pools tested positive, and amounts of DNA ranged from <500 copies/mL to >10^6^ copies/mL plasma.

Using a sequence-independent polymerase chain reaction (PCR) amplification method, we recently identified a new parvovirus in plasma from a patient with exposures and symptoms consistent with acute HIV infection, but who was HIV RNA negative (*1*). Phylogenetic analyses of sequence data suggest that this virus, termed PARV4, is only distantly related to previously known human or animal members of the family *Parvoviridae*, including members of the *Erythrovirus* genus known to infect humans, such as parvovirus B19. Infection with parvovirus B19, although frequently asymptomatic, may result in erythema infectiosum, arthropathy, pregnancy complications (e.g., hydrops fetalis), transient aplastic crisis, and disease in immunocompromised patients (*2*). Parvovirus B19 is most frequently transmitted through the respiratory route or vertically from mother to fetus. However, blood- and plasma-derived medical products, particularly clotting factors, contaminated with parvovirus B19 can also transmit the virus (*3*). Manufacturers of plasma derivatives screen minipools by using nucleic acid amplification techniques (NAT), which has enabled levels of erythrovirus DNA to be substantially reduced in start pools; for certain products, screening is now a regulatory requirement (*4*). This study examined pooled human plasma for fractionation to detect PARV4 DNA sequences.

## The Study

Samples of manufacturing plasma pools submitted to the National Institute for Biological Standards and Control for testing for hepatitis C virus RNA were stored at –70°C until analysis, in compliance with European regulatory requirements. Manufacturing pools were sourced from donations collected in Europe and North America and received during the previous 6 months. Total nucleic acid was extracted from plasma pools as described previously (*4*) before analysis for PARV4 DNA.

**Figure 1:**
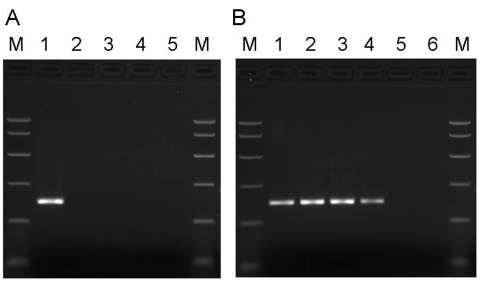
A) Specificity of primers for PARV4. Samples in lanes 1–5 were amplified by using primers directed to open reading frame 1 (ORF1) of PARV4. Template DNA in lane 1 was a plasmid subclone of the PARV4 ORF1 region. In lane 2, the template DNA was derived from parvovirus B19 International Standard (99/800, National Institute for Biological Standards and Control, South Mimms, UK) as representative of genotype 1 erythrovirus sequences; in lane 3, the template DNA was derived from a genotype 2 erythrovirus plasmid clone (A6; obtained from K. Brown, National Heart, Lung and Blood Institute, Bethesda, MD, USA); in lane 4, the template DNA was derived from a genotype 3 erythrovirus plasmid clone (D91.1; obtained from A. Garbarg-Chenon, Hôpital Trousseau, Paris, France). Template DNA in the erythrovirus samples (lanes 2–4) was adjusted to give ≈10^5.5^ copies of each genotype per reaction. Lane 5, no template control. Polymerase chain reaction (PCR) products were analyzed on a 2.5% agarose gel alongside PCR Markers (M) (Promega, Madison, WI, USA). B) Screening manufacturing plasma samples for PARV4. Samples in lanes 1–6 were amplified by using primers directed to the ORF1 region of PARV4. Template DNA in lanes 1 and 2 consisted of 1 × 10^2^ and 1 × 10^3^ copies of the ORF1 subclone of PARV4. In lane 3, the template DNA was derived from a plasma pool containing 3.9 × 10^6^ PARV4 genome copies/mL plasma; in lane 4, the template DNA was derived from a plasma pool containing <500 PARV4 genome copies/mL plasma; in lane 5, the template DNA was derived from a plasma pool that tested negative for PARV4 sequences. Lane 6, no template control. PCR products were analyzed on a 2.5% agarose gel alongside PCR Markers (M) (Promega).

**Figure 2:**
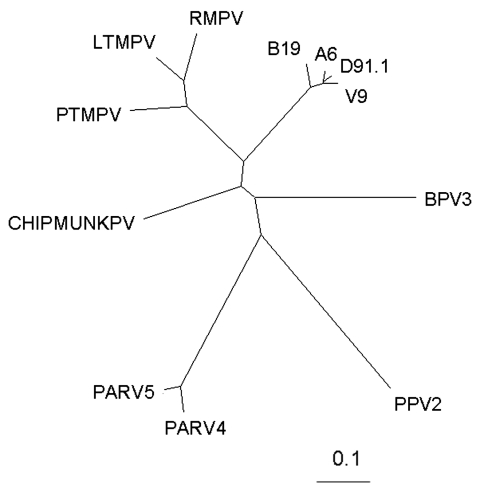
hylogenetic analysis of a 178-bp sequence of ORF1 of PARV4 and PARV5 (GenBank accession no. DQ112361) with other members of the *Parvoviridae* subfamily. The alignment includes the members of the *Erythrovirus* genus (parvovirus B19 [5]) and related viruses such as V9 (*6*), D91.1 (*7*), and A6 (*8*), as well as the closely related viruses infecting the cynomolgus macaque (LTMPV) (*9*) and rhesus (RMPV) and pig-tailed macaques (PTMPV) (*10*). Two other viruses tentatively assigned to the group include a parvovirus isolated from chipmunks (*11*); BPV3, a novel bovine parvovirus (BPV3) (*12*); and porcine parvovirus 2 (PPV2) (*13*). Analysis was performed by using the program ClustalW (*14*).

Using multiple sequence alignments of human erythroviruses and comparison with the sequence for PARV4 (*1*), we designed highly selective primers to the open reading frame 1 (ORF1) of PARV4, homologous to the nonstructural proteins of other parvoviruses. Primers PV4ORF1F (5´-AAGACTACATACCTACCTGTG-3´) and PV4ORF1R (5´-GTGCCTTTCATATTCAGTTCC-3´) amplify a 220-bp region of ORF1. The specificity of these primers was confirmed by PCR using a cloned fragment of the ORF1 region alongside erythrovirus control material (Figure 1A). Each PCR contained 1× PCR buffer II (PE Applied Biosystems, Warrington, UK), 200 μmol/L each deoxynucleoside triphosphate, 2 mmol/L MgCl_2_, 10 pmol each primer, and 2.5 U AmpliTaq Gold DNA polymerase (PE Applied Biosystems) in a final volume of 50 μL. For thermal cycling, a T3 thermal cycler (Biometra, Göttingen, Germany) was used with the following cycling conditions: 95°C for 9 min, followed by 45 cycles of 96°C for 30 s, 55°C for 30 s, and 72°C for 1 min. Amplicons were analyzed by agarose gel electrophoresis and compared to known size markers. The PARV4 control sequences (nucleotides 1293–1833 of ORF1, GenBank accession no. AY622943) were cloned into the vector pT7 Blue according to the manufacturer's instructions (Novagen, Darmstadt, Germany). The sensitivity of these PCR reactions was 1–10 copies of PARV4 sequences. DNA extracted from 137 pools was screened for PARV4 ORF1 sequences by PCR using 5 μL extracted DNA. Results, summarized in Table 1, show that 7 of 137 plasma pools screened with these primers tested positive for PARV4 DNA sequences and those of a related variant, known as PARV5. Typical results from pools and control plasmid samples are shown in Figure 1B. DNA sequence analysis showed that PARV5, over the region amplified, shares ≈92% nucleotide identity with PARV4 (Figure 2). Further sequence analysis around the primer-binding sites showed that the primers were 100% homologous in both genotypes. This level of relatedness is similar to that seen for the different erythrovirus genotypes (*7*).

**Table 1:** Analysis of plasma pools for PARV4 and PARV5

Manufacturer	No. positive/no. analyzed
A	5/12
B	0/7
C	0/9
D	2/6
E	0/14
F	0/21
G	0/50
H	0/16
I	0/2

The levels of PARV4 in the positive plasma pools were determined by real-time PCR using the screening primers from the ORF1 region of PARV4. Amplification reactions were performed on the LightCycler instrument using the LightCycler FastStart DNA Master^PLUS^ SYBR green I kit (Roche Applied Science, Mannheim, Germany) in accordance with the manufacturers' instructions. A standard curve was generated from the cloned plasmid DNA containing the ORF1 fragment of PARV4. Levels of PARV4 DNA were as high as 3.9 × 10^6^ copies/mL plasma, although several pools contained <500 copies/mL plasma (Table 2).

**Table 2:** Viral loads in plasma pools that tested positive for PARV4 or PARV5 sequences

Positive pool	Manufacturer	PARV4 viral load (genome copies/mL plasma)	Human erythrovirus viral load (IU/mL plasma)
1	A	5 × 10^5^	Negative
2	D	<500	Negative
3	A	3.9 × 10^6^*	140
4	A	<500*	340
5	A	2.1 × 10^4^*	Negative
6	A	<500*	Negative
7	D	Not determined	Not determined

*Sequences contaminating plasma pool represent PARV5 and not PARV4.

Plasma pools found positive for PARV4 sequences were tested for the levels of erythrovirus DNA as described previously (*4*). Only 2 of the PARV4-positive pools contained any human erythrovirus DNA, and these were at low levels (Table 2). Of the plasma pools found to be positive for PARV4 sequences, blood products from only 2 were available for further analysis. Both products were immunoglobulin preparations, and in neither case could PARV4 sequences be detected.

## Conclusions

This report is the first to describe novel parvovirus sequences in pooled human plasma for fractionation. PARV4 was originally identified in a patient with acute viral infection syndrome coinfected with hepatitis B virus (*1*). As yet, nothing is known about the prevalence of PARV4, its possible role in human disease, or whether PARV4 was transmitted to the original patient from an unidentified animal host.

Although PARV4 shares limited homology with human erythroviruses, the latter are frequent contaminants of plasma, pooled and used for fractionation (*3*). Levels of PARV4 DNA ranged from <500 copies/mL to >10^6^ copies/mL plasma. If a single donation with a high PARV4 count was responsible for the contamination of such a pool, the levels of virus DNA in the original donation would have been in the order of 10^9^ or 10^10^ copies/mL plasma, given the volume of the start pool. Because erythroviruses are small, nonenveloped, and relatively resistant to virus inactivation procedures, manufacturers of plasma-derived products have used NAT to exclude high-titer donations from manufacturing start pools. Before such measures were introduced, more than half of production start pools contained erythrovirus DNA, some with titers of 10^9^ copies/mL plasma (*4*; S. Baylis, unpub. data). The prevalence of PARV4 and PARV5 and the titers observed in the pools examined in this study are much lower than the usual prevalence and titers observed with erythroviruses. Because of PARV4's insufficient homology with human erythroviruses, current methods of NAT are unlikely to identify donations positive for PARV4.

The availability of highly specific reagents for PARV4 and PARV5 will assist in further studies to elucidate their possible role in human disease. The detection of PARV4 and PARV5 in plasma may have been caused by an epidemic at the time of plasma donation. In a recent study that screened for enteroviruses in human plasma, seasonal changes were observed in the frequency and level of viremia (*15*). Studies to examine the epidemiology of PARV4 and PARV5 infection will help address issues such as these.

In summary, PARV4, a novel parvovirus, and PARV5, a related variant, have been identified in plasma used in the manufacture of blood products. Plasma is obtained from healthy persons, who at the time of donation are asymptomatic, despite being viremic for PARV4 or PARV5. Highly specific and sensitive assays to detect PARV4 will facilitate further analysis of the role of this novel virus in human disease and the implications of virus transmission by contaminated blood and blood products.

## Note

After this article was submitted for publication, human bocavirus, a novel parvovirus, was identified in respiratory tract samples (*16*). PARV4 and PARV5 are distinct from human bocavirus. For example, comparison of PARV4 (AY622943) with human bocavirus strains ST and ST2 (DQ000495 and DQ000496) shows nucleotide identity of 41% and 40%, respectively.
